# Evaluating sewing operation complexity and its influence on sewing operation quality

**DOI:** 10.1016/j.heliyon.2023.e13867

**Published:** 2023-02-20

**Authors:** Huimin Li, Fansen Kong, Liang Kong, Taibao Chen

**Affiliations:** a School of Mechanical and Aerospace Engineering, Jilin University, Changchun, 130025, China; b Jilin Provincial Key Laboratory of Designing and Planning for Factory, Jilin University, Changchun, 130022, China; c Sheffield University Management School, The University of Sheffield, Conduit Road, Sheffield, S10 1FL, United Kingdom; d School of Management, Jilin University, Changchun, 130025, China

**Keywords:** Sewing operation, Action complexity, Posture keeping complexity, Perceived complexity, Cognitive complexity, Sewing defects, Fluctuation of sewing operation time

## Abstract

With the growing demand for individualization, production patterns in the garment industry now require variety and small-batch production, which has led to increasingly complicated tasks and rising workloads for sewing workers. In the present study, a new measurement method for sewing operation complexity is proposed. On this basis, the relation between sewing operation complexity and sewing operation quality is analyzed in depth. The results show that action complexity and cognitive complexity are significantly correlated with the fluctuation of sewing operation time and the rate of sewing defects. Further, there was no significant correlation between posture keeping complexity and the fluctuation of sewing operation time or between posture keeping complexity and sewing defect rate. The method proposed in this paper is helpful to better understand the complexity of sewing operations and provide theoretical guidance for quality control in sewing operations.

## 1 Introduction

Managing production in the garment industry is becoming increasingly difficult because of the requirements of multiple garment varieties, small batches, and short deadlines; subsequently, low production efficiency and high defect rates have become urgent problems for garment enterprises. Sewing is the main process, after design and cutting, which has an important impact on final product quality. The individualized characteristics of garment products make the sewing operation highly complex, while garment defects are a serious concern. Previous scholars have discussed the relationship between complexity and defect rate in depth and established prediction models of complexity and defect rate in the mechanical and electrical products manufacturing field. Kong et al.) established prediction models of the welding assembly defect rate in three dimensions: (1) complexity of product structure and defect rate, (2) assembly operation complexity and defect rate, and (3) assembly decision complexity and defect rate [1]. Shibata et al. established a prediction model of electronic product complexity and defect rate from the dimensions of design and process. They pointed out a high correlation between complexity and defect rate [2]. Su et al. established a prediction model of the relationship between assembly complexity and defect rate, and applied this model to copier products and verified it [3]. Zhu et al. noted that a higher degree of complexity corresponded to a higher defect rate under time constraints [4]. Maudgalya et al. demonstrated a significant positive correlation between ergonomic load and defect rate [5]. Bubb stated that human errors considerably influenced the quality of production processes [6]. Eklun pointed out that defect rate perators completing a task with a high ergonomic load was three times that of operators completing a general task [7].

Conversely, some scholars have shown that no significant relationship exists between complexity and defect rate. Falck and Rosenqvist divided complexity into five levels and ergonomic load into three levels according to 16 standards of complexity. The results showed that ergonomic load had a significant correlation with defect rate, whereas task complexity and defect per unit (DPU) did not show a significant correlation [8]. Falck et al. found that, in the 16 standards of complexity, only a few discriminate criteria (i.e., operating time pressure, operator skill level, and quality inspection standard threshold) had significant influences on defect rate [9]. However, the abovementioned models and methods are applied in the field of mechanical assembly and electronic product production, which require a higher standardization of products.

Conversely, the clothing industry belongs to the fast-fashion industry and clothing garments have strong personal characteristics. Thus, previous models and methods used in other fields cannot be directly implemented. However, the previous literature provides an important reference for the quantification of operational complexity in this paper. Alkan et al. proposed quantitative evaluation methods for the complexity of assembly operations based on a virtual model [10]. Zhang pointed out that information-theoretic modeling of manufacturing organizations has led to the development of measures on the information of states of manufacturing systems [11]. Deshmukh pointed out that information entropy as a commonly used method to analyze cognitive complexity [12]. Kong considered that, from the perspective of information processing economic principles, cognitive complexity was determined by the amount of information contained in the operation and the transfer relationship between information. He further measured the cognitive complexity of welding assembly workers by using the information entropy method [13]. Ahmed et al. previously reported that the sewing procedure used to assemble garment parts together is the most labor intensive stage in the garment manufacturing system [14]. To evaluate the sewing workers and workshops, standard working hours are set for the various sewing procedures, wherein industrial engineering has been used to determine these standard working hours based on various liberal factors [[15], [16], [17]]. Nchalala proposed the sewing standard allowable time (SAM) and further improved the sewing process [18]. To improve the accuracy of GSD (Garment Sewing Data) system in measuring standard working hours, on the basis of comprehensive analysis of PTS (scheduled time system) (including MOD method, MTM method and WF simple method), the time value of GSD action code is optimized [19]. However, these methods have some limitations. For example, the obtained results depend on the knowledge and experience of the analyst, and so different standard time results could be determined by different analysts. Shao et al. proposed a method for measuring the difficulty coefficient of the sewing process using the AHP method [20]. In addition, they also established a fitting curve between the standard operation time of the sewing process and the process difficulty coefficient, and predicted the standard operation time of the sewing process through the process difficulty coefficient. Yucel and Guner analyzed the influence of arrival distance, placement distance, seam length, stitch density, seam shape, fabric weight, sewing machine motor speed, fabric size and other factors on sewing time. The research results show that the influence of fabric weight and stitch density on unit time is not statistically significant, while other factors have important effects on sewing time. However, this study did not discuss the influence of human factors on sewing operation time [21]. There are few studies on the relationship between sewing operations' complexity and defect rate. Clothing products have the characteristics of popularity, novelty, and fashion; compared with the production of standardized parts, the clothing production process is more complex. It is found that there are certain differences between the actual operating time of the sewing process and the standard one, and there are always certain fluctuations. If these differences are reflected in different products and fabrics, they may be caused by factors such as materials and styles. However, the fluctuation between the actual sewing time and the standard one for the same fabric and product indicates that the sewing defect rate is mainly related to the sewing operation. This paper aims to apply the complexity analysis method to the field of garment production, evaluate sewing operations’ complexity quantitatively, and test the relationship between sewing operation complexity and quality.

In order to consider human factors, explore the methods of objectively measuring the complexity of sewing operations, and establish the relationship between the complexity of sewing operations and the fluctuation of sewing operation time and the rate of sewing defects, this paper adopts the method of information entropy to objectively evaluate the complexity of sewing operations from the four dimensions of the action complexity, the posture keeping complexity, the perception complexity and the cognition complexity. Pearson analysis was used to test the relationship between sewing operation complexity and sewing operation time fluctuation and sewing defect rate. The first part summarized and analyzed the relevant literatures on the relationship between operational complexity and complexity, defect rate and operational time. The second part put forward assumptions based on the analysis results of relevant literatures, and puts forward the evaluation methods of sewing operation complexity. The third part took clothing company A as a case study, and evaluation results of sewing operation complexity were given. Pearson correlation analysis was used to verify the hypothesis in the fourth part. The fifth part was conclusion and discussion.

## 2 Research methods

### 2.1 Background of case

For the purpose of this case study, Company A was used as an example. With a total construction area of 100,000 m^2^, more than 1100 employees, and eight subsidiaries, Company A is a brand clothing enterprise that integrates design, production, and sales, with total assets of ∼400 million CNY. Company A has workshops aimed at women's wear, trousers, suits, casual wear, and shirt production. Furthermore, Company A boasts an annual production of ∼4 million pieces. Women's windbreakers produced in workshop A were considered as an example to evaluate manufacturing complexity of clothing products.

### 2.2 Selection of samples

**Fig. 1:**
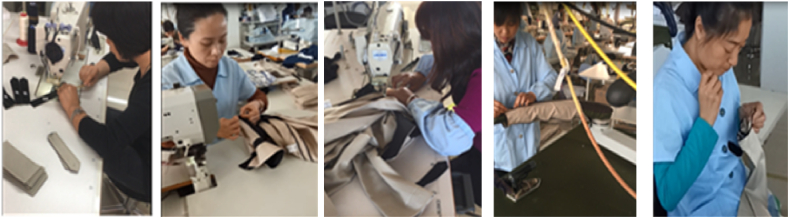
Video data of partial sleeve sewing operation.

Because there are many styles of women's clothing and the production process is more complex, women's clothing was used as an example in this paper. The production process of women's clothing includes sewing of the first samples of the garment, as well as the preparation, front-pieces, collar-sewing, sleeve-setting, back, synthesis, and pressing processes. Each process consists of dozens of subprocesses as shown in Table 1. Taking the sleeve-setting process as an example, some sewing processes are shown in Fig. 1. Regardless of whether sewing is performed using a machine or by hand, individuals must be directly involved in the process and possess certain sewing operation skills. To explore the relationship between sewing operations' complexity and quality, while controlling for the human factor, we selected the second group for on-site investigation in the women's clothing workshop of company A. There were 7 workers in the second group and these workers were standard worker. We explained the purpose of the investigation to the sewing workers. These sewing workers had sewing skills to mitigate the impact of work experience on sewing operation quality. A single windbreaker design was selected to eliminate the influence of different style design factors and sewing operation differences.

**Table 1:** Summary of the production process of women's windbreakers.

No.	Process flow	Key process	Related equipment
1	Sewing first sample	Trial sewing, inspection, sealing sample	Sewing machines, special machines, instruments for pressing
2	Preparation process	Subcontracting, trademark fabric relaxing, zipper fabric relaxing	Workbench
3	Front pieces process	Sewing pocket, shoulder piece, sewing front edge, hide thread end	Sewing machines, special machines, instruments for pressing
4	Sleeve sewing process	Attaching cuff to sleeve, top stitching for sleeve, tacking sleeve tab, press sleeve tab, top stitching to sleeve tab, hide thread end, buttonhole;	Sewing machines, special machines, instruments for pressing
5	Collar sewing process	Sewing collar, press collar, blocking sewing bound collar, sewing collar outer, top stitching for collar, hide thread end;	Sewing machines, instruments for pressing
6	Back sewing process	Split joint, top stitching	Sewing machines
7	Synthesis process	Joining shoulder seam, press shoulder, setting sleeves, attaching collar, top stitching for armhole, piping armhole, attaching zipper, setting hangtag loop, hide thread end	Sewing machines，special- machines，instruments for pressing
8	Pressing process	Press collar, press sleeve, press shoulder, press pocket	Instruments for pressing

### 2.3 Data acquisition

Data acquisition method: on-site video recording was conducted in the sewing workshop A, and then analyze the video data. In the process of Site investigation, the authors paid attention to observing the sewing worker's motions to ensure that the sewing workers completed the sewing operation in a good and stable emotional state, to eliminate the influence of negative emotions and other factors on their operation. The authors tried not to interfere with sewing workers' activities when conducting on-site research, to eliminate any psychological pressure workers could experience. Data on sewing operation time fluctuation and sewing defect rates were obtained from an on-site investigation and from the quality management department of company A. Taking the sewing of a windbreaker as an example, this study examined the defect rate across the 96 main sewing processes performed by 7 sewing workers in a women's clothing workshop from June to September 2021. The time fluctuation of the sewing process is expressed by the variance between the time of the actual operation and the standard operation time. Since the workshop is a baling operation, the operations were repeated 10 times, and the actual completion time of each process was recorded 10 times; subsequently, the average value was obtained.

### 2.4 Evaluation method of the physical-based complexity factor in sewing operations

#### 2.4.1 Evaluation method for sewing action complexity

Clothing production involves design, cutting, sewing, and inspection. Sewing itself is the main process; accordingly, it is a key factor in the quality of the final clothing product. This section presents the sleeve-setting process as an example to analyze and evaluate the physical-based complexity factor based on the modular arrangement of predetermined time standards (MODAPTS) [22]. This method divides actions into three types: move action, terminal action, auxiliary action. A video was recorded at the company's site (company A).

Sewing is a light physical operation with a general physical load lower than 2 kg. Therefore, L1 (the motion that considering the weight factor) was omitted; And rotation motion was not used in the sewing operations, therefore, C4 (rotation action)was also omitted. Then actions were classified into 19 items categories on MODAPTS, as shown in Table 2.

**Table 2:** Basic action classification of sewing operation based on MODAPTS.

No.	Type	Denotation	MODs	Description
1	Move action	M1	1	Finger movement
2	Move action	M2	2	Wrist movement
3	Move action	M3	3	Forearm movement
4	Move action	M4	4	Back arm movement
5	Move action	M5	5	Straight arms
6	Move action	W5	5	Walk
7	Move action	B17	17	Bend
8	Move action	S30	30	Get up and sit down
9	Move action	F3	3	Pedal action
10	Terminal action	G0	0	Touch
11	Terminal action	G1	1	Simple grasp
12	Terminal action	G3	3	Complex grasp (need attention)
13	Terminal action	P0	0	Simple placement
14	Terminal action	P2	2	Complex placement (need attention)
15	Terminal action	P5	5	Assemble (need attention)
16	Auxiliary action	E2	2	Visual
17	Auxiliary action	R2	2	Adjust
18	Auxiliary action	D3	3	Judgment and response
19	Auxiliary action	A4	4	Press down

Then MODAPTS method was used to analyze the motion of the kth sewing operation. According to the method of information entropy, the physical-based complexity of the kth sewing operation was calculated using equations (1)–(3) as follows:

(1) $$C_{M_{ij}}=-\sum_{j=1}^{3}\sum_{i=1}^{n_{j}}P_{ij}\;\log_{2}P_{ij}$$

(2) $$P_{ij}=\frac{M_{ij}}{M_{j}}$$

(3) $$M_{j}=\sum_{i=1}^{n_{j}}M_{ij}$$

where j represents the type of action (j = 1, represents move action; j = 2, represents terminal action; j = 3 represents auxiliary action). Mij represents the time value of the ith motion of the jth type action; nj represents the quantity of motion types in the jth type action.

#### 2.4.2 Evaluation method for posture keeping complexity

**Fig. 2:**
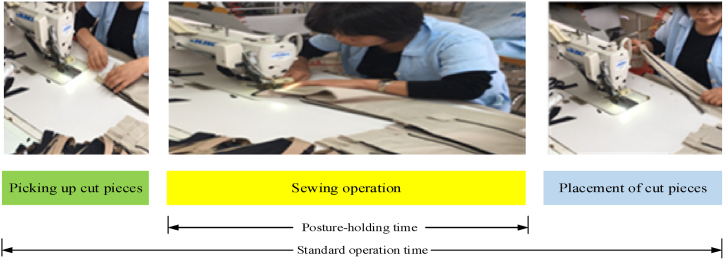
Standard operation flow of sewing operation.

Sewing workers remain in a sitting position for several hours, using their hands to sew garments manually or operate sewing machines, which results in fatigue. In practice, sewing workers are in an intermittent static working state. Sewing workers' standard operation process is divided into three stages: preparation (which implies picking up cut pieces), posture-holding time (which requires workers to assume a static position during the sewing operation), and placement (where cut pieces are arranged), as shown in Fig. 2. While the sewing posture is maintained, sewing workers' actions remain basically unchanged. During the preparation and placement stages, workers' activities are relatively varied, while their posture changes constantly. Sewing workers’ probability of posture-holding while completing a task can be expressed as the ratio of their static holding time to the standard operation time. According to the information entropy theory, the posture keeping complexity of the kth sewing operation of sewing workers is quantified as:

(4) $$C_{PK}=\sum_{K=1}^{3}P_{K}\log_{2}P_{K}$$

(5) $$P_{K}=\frac{T_{K}}{T}$$

where *T*_*k*_ is static holding time of the kth sewing operation, T is the standard operation time of the kth sewing operation.

### 2.5 Evaluation method of information processing complexity in sewing operations

#### 2.5.1 Evaluation method for perceived complexity

**Fig. 3:**
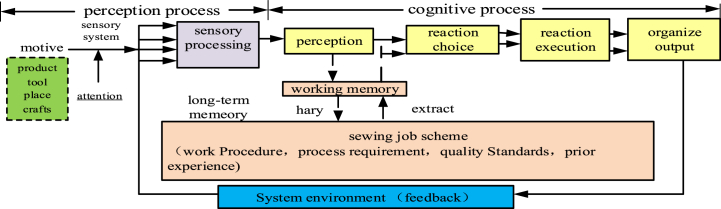
Sewing operation information processing process model.

Essentially, the human cognitive process comprises information processing. Bishu and Drury noted that the more information was obtained by an operator, the higher their error rate [23]. Based on the information processing model [24], this study proposes a model for sewing operations. Sewing workers' cognitive behavioral process involves information acquisition, processing, and output; Fig. 3 shows sewing workers’ cognitive process.

Due to the increase in the amount of information and the relationship of information in recent decades, sewing workers' uncertainty when trying to obtain useful information increases. In addition, such uncertainty increases the information processing-based complexity of sewing operations. This section adopts the information entropy method to evaluate the perceived complexity factor from three aspects: (1) information input process, (2) information processing process, and (3) information output process. The information input process is described as a perception process within sewing operations. The complexity of sewing workers' information processing was quantified according to Kong's methodology [13], in which perceived complexity is calculated as:

(6) $$H\left(X\right)=-\sum_{j=1}^{4}\sum_{i=1}^{n_{j}}p_{ij}\;\log_{2}p_{ij}$$

where *n*_*j*_ indicates the number of the information category, while *P*_*ij*_ is the ratio of the sum of the *i*_*th*_ information relationship in the j_th_ category, and the sum of the j_th_ category in the information relationship.

#### 2.5.2 Evaluation method for cognitive complexity

The information processing and output processes were described as part of the sewing operation cognitive process. Information processing is the core of sewing workers' cognitive process. According to Kong's methodology [13], the complexity of information processing is presented as follows:

(7) $$\begin{array}{l} H\left(I\right)=-\sum_{i=1}^{n}p_{i}\log_{2}p_{i}=-\sum_{i=1}^{n}\frac{f_{i}}{F}\times \log_{2}\frac{f_{i}}{F} \end{array}$$

where *f*_*i*_ is the number of occurrences of any production information *i* in the valid information chain, i.e., the frequency of production information *i* appearing in the brain during information processing. In addition, *F* is the total amount of valid information involved in all information chains.

Information output is the last stage in the cognitive process of sewing workers. According to Kong's methodology [13], the information output complexity is presented as follows:

(8) $$H\left(M\right)=-\sum_{i=1}^{n}p_{i}\log_{2}p_{i}=-\sum_{i=1}^{n}\frac{m_{i}}{M}\times \log_{2}\frac{m_{i}}{M}$$

where *m*_*i*_ is the amount of information contained in each valid information chain *i*, and *M* is the total amount of information output. *C*_*ri*_ is used to represent the cognitive complexity of the sewing operation, which is depicted as:

(9) $$C_{ri}=w_{1}H\left(I\right)+w_{2}H\left(M\right)$$

where $w_{i}$ is chosen between (0, 1) according to the actual scenario.

### 2.6 Hypothesis

These operations’ complexity is divided into physical-based and information processing-based. Physical complexity is further divided into action and posture keeping complexity, whereas information processing-based complexity is further divided into perceived complexity and cognitive complexity. Operation quality is measured by operation time fluctuation and sewing defect rate. *Sewing action complexity, posture keeping complexity, perceptual complexity*, and *cognitive complexity* are regarded as independent variables. Further, *sewing operation time fluctuation* and *defect rate* are regarded as dependent variables. To explore this issue, the following hypotheses were developed:

H1a A correlation exists between action complexity and the fluctuation of sewing operation time.

H1b A correlation exists between posture keeping complexity and the fluctuation of sewing operation time.

H1c A correlation exists between perception complexity and the fluctuation of sewing operation time.

H1d A correlation exists between cognitive complexity and the fluctuation of sewing operation time.

H2a A correlation exists between action complexity and sewing defect rate.

H2b A correlation exists between posture keeping complexity and sewing defect rate.

H2c A correlation exists between perception complexity and sewing defect rate.

H2d A correlation exists between cognitive complexity and sewing defect rate.

## 3 Case study

### 3.1 Evaluation results of the physical-based complexity factor in sewing operations

#### 3.1.1 Evaluation results of action complexity

The following analysis considers the sleeve-setting process as an example. Setting sleeves in requires suturing the sleeves to the top and arc of the armhole. To fit the three-dimensional shape of human body, the crest of the sleeve top is slightly longer than the armhole. The sleeve top and armhole are two related structural lines; to guarantee an even sleeve, the sleeve top and armhole must be accurately aligned. Table 3 lists the sleeve-setting process and quality inspection requirements.

**Table 3:** Sleeve technique and quality inspection requirements.

Process	Picture	Process requirements	Inspection requirements
**Alignment**		Sleeve top and armhole must be accurately aligned	1. Appropriate front and rear sleeves, over half of the cuff cover, and two sleeves symmetrical.
**Suture**		Sleeve top and armhole are even and appropriate	2. Sleeve top and armhole matched well.
**Remove thread**		Remove the thread	3. Armhole and lining of shoulders are suitable.
**Inspection**		Check silhouette of sleeve	4.Sleeve and bottom are smooth.

The MODAPTS method is used to analyze the picking and positioning stages of sleeve-setting, Table 4 summarizes the findings of the MODAPTS analysis for the alignment operation. According Table 4, all 19 actions of sewing operation are listed in Table 5, while the action complexity of the alignment operation is calculated using Equations (1), (2), (3)), and the Calculation results is 5.2054，as equation (10), (11), (12), (13)). The MODAPTS method is also used to analyze the inspecting stages of sleeve-setting, Table 6 summarizes the findings of the MODAPTS analysis for the inspection operation. According Table 6, all 19 actions of sewing operation are listed in Table 7, while the action complexity of the inspection operation is calculated using Equations (1), (2), (3)), and the Calculation results is 3.2890，as equation (14).

(10) $$P_{21}=\frac{M_{21}}{M_{1}}=\frac{6}{63}=0.0952$$

(11) $$C_{M_{21}}=-P_{21}\;\log_{2}P_{21}=0.3169$$

(12) $$C_{M_{i1}}=-\sum_{i=1}^{9}P_{i1}\;\log_{2}P_{i1}=2.4896$$

(13) $$C_{M_{ij}}=-\sum_{j=1}^{3}\sum_{i=1}^{9}P_{ij}\;\log_{2}P_{ij}=5.2054$$

**Table 4:** MODAPTS analysis of the alignment operation on setting in sleeve process.

**No.**	**Work step**	**Code**	**MODs**	**Frequency**
1	Lifting sleeves and semi-finished clothes from the workbench	M4G1	5	1
2	Move sleeves and semi-finished clothes to sewing worker's front	M4P2	6	1
3	Put sleeves into the semi-finished clothes	M5P2	7	2
4	Remove the thread	M4(G3*2)P0	10	1
5	Raise sleeves and align cuffs and armholes	M3G3	6	1
6	Reconfirm the alignment	M2E2D3	7	3
7	Move cuffs and armholes to the sewing machine	M3	3	1
8	Reconfirm the alignment	E2D3	5	3
9	Move sleeves and semi-finished clothes to sewing machine	M3P2	5	1
10	Step on the pedal to move the needle up	F3W5	8	1
11	Put cuffs and armholes under the needle	M3P5	8	1
12	Step on pedal, put the needle down	F3	3	1
**13**	**Hold the cuffs and armholes at the junction**	**M4P5**	**9**	**1**

**Table 5:** The probability of 19 actions on alignment operation.

NO.	Action I	Frequency	*P*_*ij*_	NO.	Action II	Frequency	*P*_*ij*_	NO.	Action III	Frequency	*P*_*ij*_
1	M1	0	0	10	G0	0	0	16	E2	12	0.4000
2	M2	6	0.0952	11	G1	1	0.0357	17	R2	0	0
3	M3	12	0.1905	12	G3	9	0.3214	18	D3	18	0.6000
4	M4	16	0.2540	13	P0	0	0	19	A4	0	0
5	M5	10	0.1587	14	P2	8	0.2857				
6	W5	10	0.1587	15	P5	10	0.3571				
7	B17	0	0								
8	S30	0	0								
9	F3	9	0.1429								
$\sum_{i=1}^{n_{j}}M_{ij}$		63	1			28	1			30	1

**Table 6:** MODAPTS analysis of the inspection operation on setting in sleeve process.

No.	Work step	Code	MODs	Frequency
1	Pick up clothes with both hands	M5*2P2E2D3	17	1
2	Check the appearance of the sleeves	M4P5E2D3	14	1
3	Review repeatedly	M4*2E2D3	13	1
4	Walk to the model	M4P2W5*5	31	1
5	Hang the clothes on the model	4M4P5	9	4
6	Adjust clothes and check	8M3P5E2	8	8
7	Lift the left hand, adjust the clothes, place the clothes again, adjust	M5M3P5E2D3	18	1
8	Lift the right hand, adjust the clothes, and fix the clothes with the left hand	3M4P5E2D3	14	3
9	Complete the inspection and remove the clothes	M5P2	7	1

**Table 7:** The probability of 19 actions on inspection operation.(14)$C_{M_{ij}}=-\sum_{j=1}^{3}\sum_{i=1}^{9}P_{ij}\;\log_{2}P_{ij}=3.2890$

NO.	Action I	Frequency	*P*_*ij*_	NO.	Action II	Frequency	*P*_*ij*_	NO.	Action III	Frequency	*P*_*ij*_
1	M1	0	0	10	G0	0	0	16	E2	15	0.7500
2	M2	0	0	11	G1	0	0	17	R2	0	0
3	M3	9	0.3103	12	G3	0	0	18	D3	5	0.2500
4	M4	11	0.3793	13	P0	0	0	19	A4	0	0
5	M5	4	0.1379	14	P2	3	0.1429				
6	W5	5	0.1725	15	P5	18	0.8571				
7	B17	0	0								
8	S30	0	0								
9	F3	9	0								
	$\sum_{i=1}^{n_{j}}M_{ij}$	29	1			28	1			30	

#### 3.1.2 Evaluation results of posture keeping complexity

From the operation videos recorded at each station, we find that workers are in an intermittent static working state. Taking the collar-sewing process as an example, the standard operation time, preparation time, static posture-holding time, and placement time of the main sewing process are shown in Table 2. Sewing workers remain in a seated position during the stages of preparation and placement; however, their bodies can be relatively relaxed and active. Nevertheless, during the posture-holding period, workers maintain a static posture for a long time. The posture keeping complexity was evaluated using Equations (4), (5)), the calculation results are shown in Table 8. And the posture keeping complexity evaluation result is as equation (15).

(15) $$C_{PK}=\sum_{K=1}^{3}P_{K}\log_{2}P_{K}=1.007$$

**Table 8:** Operation time of sewing process（s）.

Sewing process	picking preparation time	static holding time	placing the cutting pieces time	the standard operation time
Collar adding	12	159	39	210
Pk	0.0571	0.7571	0.1857	1

### 3.2 Evaluation results of the information processing-based complexity in sewing operations

#### 3.2.1 Evaluation results of perceived complexity

The information entropy method was used to evaluate the information input, processing, and output processes of sewing operations, taking sleeve-setting as an example. Table 9 lists the production characteristics of sleeve-setting and the resources required.

**Table 9:** Description of the setting in sleeve process.

Classification	Element	Description
Production characteristics	System	Sewing
	Part	Sleeve, Semi-finished clothes
	Process	Setting in sleeve
	Procedure	Alignment, Remove thread, Suture, Inspection
Resources required	Material	Sleeve (one piece), Semi-finished clothes (one item),Thread
	person	Sewing worker (one person)
	Tool	Sewing machine, Scissors, Mannequin
	Workplace	Bench, Mannequin's area

**Fig. 4:**
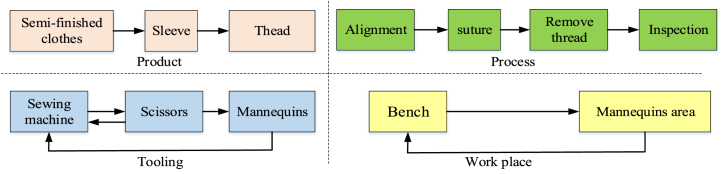
Information relationship diagram of setting in sleeve process.

Fig. 4 shows the four product information variables in the information acquisition phase of sleeve-setting, including three product variables, *X1* (*semi-finished clothes*, *sleeves*, and *thread*); four process variables, *X2* (*alignment*, *suture*, *remove thread*, and *inspection*); and three tooling variables, *X3* (*sewing machine*, *scissors*, and *mannequins*). Furthermore, two workplace variables, *X4* (*workbench* and *mannequin area*), were considered. Fig. 3 illustrates the four types of production information relationships.

The relationship matrix can be obtained from the information relationship diagram (shown in Fig. 3) presenting information of the products, processes, tools, and workplaces involved. According to Fig. 3, the complexity of the information input is the sum of the four complexities of production information. Table 8 lists the information variable relationship matrix and the input complexity factor result of the product information variables (*X1*). The method for calculating the complexity factor of the other production information variables (*X2, X3, X4*) is the same as that for *X1*. Therefore, the perceived complexity was evaluated using Equation (6), as shown in Table 10. And the calculation result of perceived complexity is as equation (16).

(16) $$H\left(X\right)=-\sum_{j=1}^{4}\sum_{i=1}^{n_{j}}p_{ij}\;\log_{2}p_{ij}=1.584+1.984+1.52+1=6.088$$

**Table 10:** Input complexity result of product information variables.

Variables	Relationship r = (1, 0)			Subtotal	$p_{i}$	$-p_{i}\;\log_{2}p_{i}$
	Semi-finished clothes	Sleeve	Thread			
﷐ Semi-finished clothes	1	1	1	3	0.333	0.528
Sleeve	1	1	1	3	0.333	0.528
Thread	1	1	1	3	0.333	0.528
Total				9	1	1.584

#### 3.2.2 Evaluation results of cognitive complexity

Taking sleeve-setting as an example, Fig. 4 shows the four valid information chains used for sleeve-setting. The total amount of information output is *M* = 21. The sleeve-setting process contained *I* = 12 types of information. The attention required for each type of information in the output process (in the four types of information) is *p*1 = *p*2 = 4/21 = 0.190, *p*3 = 2/21 = 0.095, *p*4 = *p*5 = *p*6 = *p*7 = *p*8 = *p*9 = *p*10 = *p*12 = 1/21 = 0.0476, and *p*11 = 3/21 = 0.143. The complexity factor of information processing can be calculated using Equation (7), and the calculation results is as equation (17).

(17) $$H\left(I\right)=-\sum_{i=1}^{I}p_{i}\;\log_{2}p_{i}=2.983$$

**Fig. 5:**
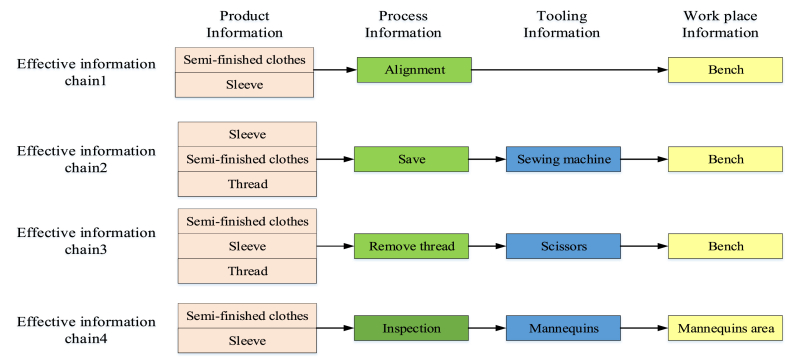
Effective information chain of the setting in sleeve operation.

The total amount of information output is *M* = 21, according to the effective information chain of the actual production process in Fig. 5. The amount of information contained in each valid information chain *i* is *m*1 = 4, *m*2 = 6, *m*3 = 6, and *m*4 = 5, while the information rate in each effective information chain is *p*1 = 4/21 = 0.190, *p*2 = 6/21 = 0.286, *p*3 = 6/21 = 0.286, and *p*4 = 5/21 = 0.238. The complexity factor of the information output process, based on Equation (8), and the calculation results is as equation (18).

(18) $$H\left(M\right)=-\sum_{i=1}^{4}p_{ij}\;\log_{2}p_{ij}=1.979$$

Based on the abovementioned analysis, the processes of information acquisition, processing, and output are equally important in the sewing process. In the sewing operation process, without loss of generality, we assume that the processes of information acquisition, processing and output are equally important for sewing workers. Therefore, the weight value is 1. The cognitive complexity is presented based on Equation (9), and the calculation results are as equation (19).

(19) $$C_{ri}=H\left(I\right)+H\left(M\right)=2.983+1.979=4.962$$

## 4 Pearson correlation analysis

Sewing operation complexity comprises sewing action complexity, sewing posture keeping complexity, sewing workers' perceptual complexity, and sewing workers' cognitive complexity, which were taken as independent variables. The 96 main sewing processes were evaluated according to the aforementioned methodology. Sewing operation time fluctuation and sewing defect rate were taken as dependent variables through Pearson correlation analysis. When the correlation coefficient is greater than 0.4 and the p value is less than 0.05, it indicates that there is a high correlation between variables. Pearson correlation analysis results are shown in Table 11.

**Table 11:** Correlation analysis between sewing operation complexity, operation time fluctuation and defect rate.

Variables	Operation time fluctuation	Sign.	Defect rate	Sign.
Motion complexity	0.654^a^	0.000	0.501^a^	0.000
Posture keeping complexity	0.267	0.009	0.227	0.026
Perceived complexity	0.251	0.014	0.284	0.005
Cognitive complexity	0.577^a^	0.000	0.415^a^	0.000

a Significant at a 0.01 level (two-sided).

## 5 Conclusion and discussion

To objectively measure the operation complexity of sewing workers, this paper mainly considers the human factor, A new method to measure the complexity of sewing operations was proposed. It measures the complexity of sewing operations from two aspects: physical-based complexity and information processing-based complexity. Hypotheses H1a–H2d were verified via Pearson correlation analysis. As shown in Table 1, Table 11 The correlation coefficient between operation complexity and operation time fluctuation was 0.654, greater than 0.4, and p-value Was 0.000; The correlation coefficient between action complexity and sewing defect rate was 0.501, greater than 0.4, and p-value was 0.000; thereby confirming H1a and H2a. (2) The correlation coefficient between attitude maintenance complexity and operation time fluctuation was 0.267, less than 0.4, and p-value was 0.009; it was greater than 0.005, the correlation coefficient between posture keeping complexity and sewing defect rate was 0.227, less than 0.4, and p-value was 0.026; it was greater than 0.005, H1b and H2b were not confirmed. That is, there is no significant correlation between the posture keeping complexity of sewing workers and the fluctuation of operation time and sewing defect rate. (3) The correlation coefficient between perceived complexity and operating time fluctuation was 0.251, less than 0.4, and p-value was 0.014; it was greater than 0.005, the correlation coefficient between attitude maintenance complexity and sewing defect rate was 0.284, less than 0.4, and p-value was 0.005. H1c and H2c were not confirmed. That is, there is no significant correlation between the perceived complexity of sewing workers and the fluctuation of operation time and sewing defect rate. (4) The correlation coefficient between cognitive complexity and operation time fluctuation was 0.577, greater than 0.4, p-value is 0.000, less than 0.005. The correlation coefficient between cognitive complexity and sewing defect rate was 0.415, greater than 0.4, p-value was 0.000, less than 0.005. Thereby confirming H1d and H2d.There is a significant correlation between cognitive complexity and fluctuation of operation time and sewing defect rate.

In previous studies, MTM or MOD methods were adopted to analyze the actions of the sewing process, and some allowance factors were considered to measure the standard operating time of the sewing process. On the basis of analyzing the actions of the sewing process with the MOD method, this paper further analyzes the types and quantities of actions in the sewing process, and evaluates the action complexity of the sewing process with the method of information entropy, which further enriches the theory of action complexity. The action complexity is related to the quantities and types of actions. Different materials and clothing styles will have an influence on the action complexity. For different materials, some actions may increase or decrease accordingly in the sewing process. For different clothing styles, the sewing length will also change accordingly, which will make the types and quantities of actions in the sewing process change. When the material or style changes, it is necessary to use the MOD method to re-analyze the sewing action and evaluate the action complexity. Meanwhile, the more operations involved in the sewing process, the more the quantities and types of corresponding actions, and the greater the action complexity. Different materials will also affect the posture keeping complexity of sewing workers. For different materials or styles, the sewing workers' static operating time may change, and it is also necessary to re-measure the static operating time and evaluate the posture keeping complexity. Previous literature proposed a method to objectively measure workers' perceptive and cognitive complexity and mainly applied this method to the field of assembly. This paper further applies this method to the field of clothing production, which expands the application of this method. According to the human information processing model, products with the same style and different materials have the same perceptive and cognitive process. Therefore, different materials have no influence on perceptive and cognitive complexity. However, clothing products with different styles have a different perceptive and cognitive process, and their perceptive and cognitive complexity is different. In actual operation, the actual operation time of sewing workers is difficult to be consistent with the standard one, and there is a certain range of fluctuations. To explore the relationship between the complexity of sewing operation, the fluctuation of actual sewing operating time, and the rate of sewing defects from the perspectives of human factors, and to avoid the influence of materials and sewing workers' skill levels on the rate of sewing defects and the fluctuation of actual sewing operating time, this paper selects the sewing process of the windbreaker with the same style and fabric for research. The selected sewing workers all have more than 5 years of experience. The research shows that the action and perception complexity has a significant influence on the fluctuation of actual sewing operating time and sewing performance.

This research has some limitations, which also reveal future research directions. When considering the complexity evaluation of the sewing operation, sewing defect rate, and the fluctuation of actual sewing time, it is only measured from the human factor. Besides, other factors may also affect the fluctuation of sewing operating time and sewing defect rate, such as clothing fabrics, personnel's skill level, sewing length, etc. Future research can focus on the multi-dimensional analysis of the time fluctuation of the clothing sewing process and the influencing factors of the sewing defect rate. This method is mainly applicable to the field of manual work, especially to the field of clothing. Future research can also consider the practical application of this method in other industries, so as to promote the universality of this research method.

## Author contribution statement

Huimin Li: Performed the experiments; Wrote the paper.Fansen Kong: Conceived and designed the experiments; Contributed reagents, materials, analysis tools or data.Liang Kong: Complexity analysis, Papers review and Polishing the manuscript; Taibo Chen: Analyzed and interpreted the data.

## Funding statement

Fansen Kong was supported by Project for Science and Technology Development of 10.13039/501100011789Jilin Provincial Department of Science and Technology [2020122355JC].

## Data availability statement

The authors do not have permission to share data.

## Declaration of competing interest

The authors declare that they have no known competing financial interests or personal relationships that could have appeared to influence the work reported in this paper.
